# Effect of kaolin on productivity, anatomical and biochemical responses to water deficit in *Pelargonium graveolens* grown in sandy soil

**DOI:** 10.1186/s12870-024-05814-x

**Published:** 2024-11-22

**Authors:** Eman F. AbuEl-Leil, Mohamed A. E. AbdelRahman, S. F. Desoukey

**Affiliations:** 1 Medicinal and Aromatic Plants Research Department, Horticulture Research Institute (HRI), Agricultural Research Centre (ARC), Cairo, Egypt; 2 Division of Environmental Studies and Land Use, National Authority for Remote Sensing and Space Sciences (NARSS), Cairo, 1564 Egypt; 3 Agricultural Botany Department, Faculty of Agriculture, Cairo University, Giza, Egypt

**Keywords:** Anatomy, Enzymes activity, Essential oil, Evapotranspiration for stander crop (ET_o_), Geranium, Kaolin, Water deficit, WUE

## Abstract

The objective of this study was to examine the response of geranium plants to different irrigation levels (100%, 80%, and 60% based on ET_o_) and Kaolin application rates (0, 100, 200 and 300 ppm) during 2022 and 2023 seasons, at Aly Mobarak Experimental Farm, Horticulture Research Station, located at El-Bustan site, El-Behiera Governorate, Egypt, by using a two-way factorial analysis experimental design. The results revealed that water deficit significantly reduced most studied traits. Irrigation level at 60% based on ET_o_ exhibited poorest performance on growth parameters and decreased fresh yield and essential oil yield by 27.77% 10.73%, respectively as compared with full irrigated plants. However, foliar application of kaolin at 200 and 300 ppm led to increasing biomass accumulation by 28.51, 26.16%, and essential oil yield by 79.51, 89.95%, respectively, as compared with untreated plants grown under the same level of water deficit (60% based on ET_o_). GC–MS analysis of essential oil showed that water deficit and kaolin application increased geraniol/citronellol ratio and consequently improved oil quality. Results highlight the positive influence of water deficit and kaolin rates on the development and performance of anatomical parameters. Enzymes assay in leaves revealed in an increase superoxide dismutase (SOD) and peroxidase (POD) activities, and decreased in catalase (CAT) activity under water deficit. As for WUE at 60%, followed by 80% based on ET_o_ recorded excellent response for geranium plants which led to more water saving. So, it could be concluded that foliar application of kaolin at 200 and 300 ppm obtained the optimal characteristics of geranium plants under experimental conditions. In particular, essential oil yield and productivity.

## Introduction

Geranium (*Pelargonium graveolens*) herbaceous plant belongs to the *Geraniaceae* family and is a considerable medicinal and aromatic plant in Egypt. It is a main source of essential oil used in many aspects, such as perfumery and food processing [1]. The major constituents of this oil are citronellol, geraniol, iso-metone, citronellyl formate, and geraniol formate [2]. Geranium oil quality is controlled by the citronellol and geraniol ratio of C/G (1–3). The lower ratio is an indication of good-quality oil [3, 4].

Water is among the most vital variables influencing growth, yield, and quality of medicinal and aromatic plants since its shortage and scarcity may cause genuine growth hurts and yield loss. Development and essential oil produced from geranium plants are negatively impacted by drought [5]. Several shortages of water affect physiological functions such as leaf development, gas exchange, and carbon fixation at the cellular level [6]. Also, it increases leaf thickness and changes leaf anatomy as well as the arrangement of both palisade and sponge tissue cells with increasing intercellular space [7]. Water stress causes stomatal closure and increases photorespiration, leading to oxidative damage due to the accumulation of reactive oxygen species (ROS) in plants [8]. ROS stress causes disruption of chloroplast leading to chlorophyll loss [9]. Plants have a defense mechanism against ROS through the induction of enzymatic and non-enzymatic antioxidant defense chemicals [10, 11]. These endogenous anti-drought compounds are inadequate to permit pushed crops to withstand water deficits. So, plants need outside application of substances that stimulate resistance [12].

Kaolin can act as a diluting factor in minimizing water stress in this connection [13] reported that kaolin mitigated the harmful effects of combined stress in several ways. Kaolin (an aluminum phyllosilicate), when applied to plants it forms thin nanoparticle films that lower the canopy temperature [14]. Furthermore, the findings demonstrated that foliar spraying kaolin on *phaseolus vulgaris* L. leaves in conjunction with skipping one DI-vegetative or DI-ripen irrigation had a positive effect on the chemical contents of leaves (N, P, K, chlorophyll, carotenoids, and TSS), plant water status (relative water content (RWC) and membrane stability index (MSI)), pods (N, P, chlorophyll, carotenoids, and TSS), and fruit firmness. which are required for plant growth. Consequently, kaolin enhanced the activities related to photosynthesis [15]. Furthermore, the activity of stress enzymes was regulated by kaolin to give maize plants the best defense against drought stress Furthermore, it has been shown that kaolin enhanced post-harvest quality, yield, color, gas exchange, photosynthetic rate, and net CO_2_ assimilation in olive, sweet basil*, Mentha pulegium, Ocimum basilicum* L, walnut, apple, mango, pomegranate, grape, tomato, and *phaseolus vulgaris* [16].

The present study was carried out to evaluate the role of kaolin rate in mitigating the effect of water deficit on geranium plants grown under sandy soil conditions.

## Materials and methods

### Experimental site and plant source

Healthy mothers of geranium are grown at Aly Mobarak Experimental Farm, Horticulture Research Station, located in the El-Bustan region, El-Behiera Governorate, Egypt. The farm is located at a latitude of 33°30′ 1.4''N, a longitude of 30°19′ 10.9''E, and an altitude of 21 m above sea level. This survey has been conducted over two consecutive seasons, 2022 and 2023.

Table 1 presents meteorological data collected on site during growth stages on both distinct seasons according to the methods described by [17].

**Table 1:** Meteorological data at the site during two seasons

**Month**		**season 2022**								
		**Feb**	**Mar**	**Apr**	**May**	**June**	**July**	**Aug**	**Sep**	**Oct**
**Temperature ℃**	**Max**	19.00	21.00	26.50	30.94	33.05	35.69	36.00	32.33	31.00
	**Min**	6.59	8.85	11.60	16.25	20.16	21.50	21.68	19.80	18.75
**RH _AVG %**		60.71	57.92	50.00	39.12	49.21	50.25	51.72	56.00	58.57
**Wind speed (m/sec)**		2.68	2.98	2.99	2.61	3.18	2.71	2.85	3.00	2.63
**Radiation (MJ m**^**−2**^**)**		14.00	18.89	24.84	26.87	28.86	28.61	26.32	22.69	17.31
**Et**_**0**_** mm day**^**−1**^		3.58	4.60	6.90	8.74	8.93	8.88	8.62	6.58	5.00
		**Season 2023**								
**Temperature ℃**	**Max**	20.90	23.90	28.50	38.04	37.95	37.79	37.72	36.93	33.58
	**Min**	9.15	10.93	13.78	18.15	22.64	23.76	23.68	22.68	20.25
**RH-AVG%**		64.25	60.06	50.82	41.00	51.95	52.73	53.84	57.28	60.79
**Wind speed (m/sec)**		2.98	3.34	3.55	3.91	3.56	3.85	3.07	3.20	3.05
**Radiation (MJ m**^**−2)**^		15.70	19.95	25.50	27.77	29.24	29.77	28.10	24.11	19.01
**Et**_**0**_** mm day**^**−1**^		3.00	4.00	5.00	6.68	7.03	7.38	6.60	6.00	3.00

**RH AVG% = relative humidity average %, (m/sec) meter per second, (MJ/m**^**2**^** day**^**−1**^**) = megajoule per square meter and per day and (mm day**^**−1**^**) millimeter per day**

Table 2 chemical and physical analyses of the experimental soil were carried out according to the methods described by [18].

**Table 2:** Physical and chemical properties of the experimental soil

	**Sandy soil**	
**Physical properties**		
	1^st^ season	2^nd^ season
Sand (%)	91.30	90.50
Silt (%)	4.60	5.60
Clay (%)	4.10	3.90
Texture	sandy	Sandy
Field Capacity, (%)	13.30	13.60
Wilting Point, (%)	4.70	4.60
Available water, (%)	8.60	9.00
Bulk density (t m^−3^)	1.79	1.78
**Chemical properties**		
EC_1:5_ (dS m^−1^)	0.75	0.53
pH (1:2.5)	8.96	8.70
Total CaCO_3_ (%)	7.00	5.66

### Experimental procedures

On 15th February 2022 and 2023, rooted terminal stem cuttings measuring approximately 10 to 15 cm in height were planted for the first and second seasons, respectively. To prepare the soil, 15 m^3^/fed. of compost and 300 kg/fed of calcium superphosphate (15.5% P_2_O_5_) were incorporated. Potassium application was carried out using potassium sulfate (48% K_2_O) at 100 kg/fed and nitrogen was applied as ammonium nitrate (33.5% N) at a rate of 600 kg/fed. This methodology involved dissolving soluble fertilizers in the fertilizer tank and applying them by fertigation. The drip irrigation system was used with a flow rate of 4 L/h. Once the plants were established, irrigation treatments were initiated at 2-day intervals. The results of the chemical analysis of irrigation water from the farm well are presented in Table 3.

**Table 3:** Chemical analysis of the irrigation water at the experimental site

pH	Ecw		Soluble anions (meq/l)			Soluble cations (meq/l)			
7.78	ppm	dS/m	CO_3_^− −^	HCO_3_^−−^	Cl^−^	Ca^++^	Mg ^++^	Na ^+^	K ^+^
	1664	2.6	-	5.20	17.20	4.00	3.60	18.01	0.32

### Irrigation levels (based on % ETO)

1. 100% of ET_o_ (3550 and 3750 m3/fed. in the first and second seasons respectively) as control,
2. 80% of ET_o_ (2840 and 3000 m3/fed. in the first and second seasons respectively) and,
3. 60% of ET_o_ (2130 and 2250 m3/fed. as in the first and second seasons respectively).

Evapotranspiration (ET_o_) mm/day was calculated by Penman Monteith equation [19] using the climatologically data of El-Bostan area according to Table 1.

Water utilization efficiency values were estimated according to [20], as follows:

$$\mathrm{Water}\;\mathrm{use}\;\mathrm{efficiency}\;(\mathrm L\;\mathrm m^{-3})=\frac{\mathrm{Oil}\;\mathrm{Yield}\;(\mathrm{liter})}{\mathrm{Water}\;\mathrm{applied}\;(\mathrm m^3)}$$

Water applied (m^3^) = Whole quantity of irrigation water for each treatment.

Oil Yield (liter) = Sum of total essential oil / fed in 1^st^ and 2^nd^ cuts for each season.

### Kaolin application

Geranium plants were subjected to two spraying cycles per cutting with kaolin particle film as a reflective at (Surround WP Crop Protectant, 95% Kaolin, 5% inner ingredients, AL-Goumhoria Co., Egypt). The first application took place during the branching initiation phase, precisely 30 days after transplanting. Subsequently, the second spraying took place at the full branch stage, i.e. 20 days after the first application. Throughout the growing season, plants were treated with different concentration levels, including 0, 100, 200, and 300 ppm.

### Harvesting

The aerial parts of the plants were cut at a height of 10–15 cm above the soil surface when they were in full flower. This harvesting process was carried out twice during each season. Specifically, the first cut took place on the 23^rd^ and 22^nd^ of May in both, respectively while, the second took place on the 22nd and 23rd of October in both, respectively.

### Data recorded

During each incision, vegetative growth characteristics were documented, including plant height in centimeters, number of branches per plant, leaf thickness in millimeters, and weight grams or plant in a fresh, dry state, measured in grams.

### Essential oil productivity

#### Essential oil extraction

The hydro-distillation method was used to extract the essential oil from freshly harvested leaves, using a Clevenger-type apparatus, following the guidelines established by [21].

#### Gas chromatography-mass spectrometry (GC–MS)

Gas chromatography and mass spectrometry (GC/MS) techniques were used to carry out the identification and analysis of essential oils. Preliminary identification of the components was performed by comparing their relative retention times and mass spectra with those stored in the NIST and WILLY libraries of the GC/MS system [22, 23].

### Anatomical study

Several investigations have been carried out on the anatomical structure of *Pelargonium graveolens L*. leaves. The microtechnology activities were carried out by Agric. Bot. Department, Faculty of Agriculture, Cairo University, Giza, Egypt. The samples underwent a series of procedures including fixation in formaldehyde, acetic acid and alcohol (F.A.A.) solution for a minimum of 48 h, dehydration and embedding in paraffin wax [24]. Subsequently, the sections, which were cut at a thickness of 15 to 20 microns using a rotary microtome, were stained with crystal violet/erythrosine before being mounted in Canada balsam. The resulting slides were then photomicrograph and observed under a light microscope.

### Determination of photosynthetic pigments contents

Pigment content was extracted by using dimethyl sulfoxide (DMSO) solvent [25]. The calculation of chlorophyll a (Chl a) and chlorophyll b (Chl b) was carried out according to the equation proposed by [26]. On the other hand, the determination of the total carotenoid concentration was calculated based on the equation described by [27].

### Determination of antioxidant activity of the enzymes

#### Sample preparation

Leaf sample (1 g) tissue was grind in the cold mortar and pestle with the addition of cold PBS (phosphate buffered saline pH 7.4, (5 – 10 ml) i,e, 50 mM potassium phosphate, pH 7.4. 1 mM EDTA and 1 ml/L Triton X-100) per gram tissue. Centrifuge at 4,000 rpm for 15 min. at 4°C. Remove the supernatant for assay and store on ice. Aliquots of 0.05 ml supernatant samples were taken for the determination of catalase and peroxidase, while 0.1 ml sample was taken for the determination of SOD enzyme [28, 29].

#### Catalase assay

- According to the method of [28], catalase activity was determined.
- Supernatant (0.05 ml) + 0.05 ml H_2_O_2_ + 0.05 ml of buffer + 100 ml diluted H_2_O.
- Incubate for exactly one minute at 25℃, then add 200 ml of chromogen- inhibitor.
- Incubate at 37℃ for 10 min read at 510 nm. against a blank sample (0.05 ml of buffer instead of plant tissue homogenate.
  $$\mathrm{CAT}\;\mathrm{activity}\;(\mathrm U\;\mathrm g^{-1})=\frac{\mathrm{standard}-\mathrm{sample}}{\mathrm{standard}}\mathrm x\frac1{\mathrm{gm}\;\mathrm{of}\;\mathrm{tissue}}$$

#### Peroxidase assay

- Supernatant (0.05 ml) + 0.05 ml diluted H_2_O + 0.05 ml chromogen.
- Incubate for 10 min. at 37℃. Read at 510 nm against blank sample using 50 ml diluted H_2_O instead of plant sample. The assay was described by [30].
  $$\mathrm{POD}\;\mathrm{activity}\;(\mathrm U\;\mathrm g^{-1})=\frac{\mathrm{sample}}{\mathrm{standard}}\mathrm X\;0.5\;\mathrm X\frac1{\mathrm{gm}\;\mathrm{of}\;\mathrm{tissue}}$$

#### Superoxide dismutase (SOD) assay

- Supernatant (0.1 ml) sample + 1 ml working reagent (R1 + R2 + R3 + in the ratio of 10 + 1 + 1 ml) use diluted H_2_O (100 ml) instead of supernatant.
- Mix well and add (R4 = PMS) at 100ml.
- Read at 560 nm for 5 min.
- R1 = phosphate buffer pH 8.5.
- R2 = Nitroblue tetrazolium (NBT).
- R3 = NADH.According to the assay designed by [31].
  $$\mathrm{percent}\;\mathrm{inhibition}=\frac{\mathrm A\;\mathrm{control}\;-\;\mathrm A\;\mathrm{sample}}{\mathrm A\;\mathrm{control}}\mathrm X100$$

$$\mathrm{SOD}\;\mathrm{activity}\;(\mathrm U\;\mathrm g^{-1})\;=\%\;\mathrm{inhibition}\;\mathrm X\;3.75\;\mathrm X\frac1{\mathrm{gm}\;\mathrm{of}\;\mathrm{tissue}}$$

### Statistical analysis

Two-way factorial analysis experimental design, three replicates were included. To analyze the significant differences observed between treatments mean, the statistical program (Statisix 8) was used to perform an analysis of variance (ANOVA). Differences between treatment means were assessed using the least significant difference (L.S.D. 0.05) test, with a probability level of 0.05, as recommended by [32].

## Results and discussion

### Effect of water deficit and kaolin rates on vegetative characteristics of geranium plants

Data in Table 4 showed the effect of foliar application of kaolin at different concentrations on growth characteristics of geranium plants grown under different water levels at both studied seasons. The results clearly showed that, plant height and shoot number gradually decreased with increasing water deficit. Maximum reduction in this trait was obtained at the water level of 60% based on ET_o_ reduction of plant height by 20.62% and shoot number by 31.80% compared with normal irrigation by 100% based on ET_o_. This could be demonstrated to dehydration had negative effects on various physiological processes in plants, such as leaf development, gas exchange at the organ level, and carbon fixation at the cellular level. Finally, normal growth and division of cells [6]. Similar results were obtained by [33] on Hot pepper and [10] on sweet basil. On the contrary, leaf thickness increased as water level decreased. It increased by 36.73% on plants irrigated with 60% These results are in agreement with [7] on *Camellia oleifera* cultivars, those reported that plant leaf thickness is increasing for more water stored is the basis for plant response and adaptation to environmental changes. Furthermore, [42] noted that, the increase in leaf thickness on 'Chemlali' cultivar was due to an increase in the thickness of the spongy parenchyma and upper palisade. That enhanced and facilitated to CO_2_ fixation and rapid diffusion of CO_2_ to these sites in plants grown under water deficit as confirmed by anatomical studies.

**Table 4:** Effect of Irrigation levels and kaolin rates on vegetative characteristics of geranium (*Pelargonium graveolens*) plants

Treatments		First Season (2022)						Second Season (2023)					
		**First cut**			**Second cut**			**First cut**			**Second cut**		
		**PH (cm)**	**NB**	**LT (mm)**	**PH (cm)**	**NB**	**LT (mm)**	**PH (cm)**	**NB**	**LT (mm)**	**PH (cm)**	**NB**	**LT (mm)**
**Irrigation levels** **(A)**	**100% (con.)**	57.5 a	10.6 a	0.82 b	65.8 a	13.8 a	0.95 b	54.3 a	14.0 a	0.88 c	62.8 a	16.4 a	0.91 c
	**80%**	53.7 a	9.1 b	0.97 a	61.2 a	12.3 a	0.99 b	46.4 b	10.9 b	0.99 b	58.3 ab	12.9 b	1.10 b
	**60%**	43.4 b	7.6 c	0.97 a	53.5 b	10.2 b	1.16 a	42.0 b	8.0 c	1.19 a	52.3 b	11.5 c	1.47a
**Kaolin rate** **(ppm)** **(B)**	**Control**	47.6 b	7.6 c	0.82 b	54.1 c	10.0 b	0.75 c	40.6 b	9.0 b	0.75 b	49.7 c	11.4 b	0.87 b
	**100**	50.8 ab	8.6 bc	0.94 a	59.6 b	11.8 ab	0.95 b	46.8 a	11.0 a	1.1 a	56.4 bc	14.1 a	1.20 a
	**200**	53.7 a	9.6 ab	1.02 a	62.0 ab	13.0 a	1.21 a	51.0 a	12.2 a	1.09 a	60.8 ab	14.8 a	1.30 a
	**300**	54.1 a	10.7 a	1.00 a	65.0 a	13.4 a	1.23 a	52.0 a	11.7 a	1.11 a	64.1 a	14.1 a	1.26 a
**Interactions** **(A X B)**	**100% + con**	53.7 ab	9.3 abc	0.69 f	60.7 bcd	12.0abc	0.65 e	44.7 de	10.7 bcd	0.60 f	54.0 cd	13.0 bc	0.71 f
	**100% + 100**	58.3 ab	11.0 a	0.83 def	65.0 ab	14.0 ab	0.93 d	55.3 abc	15.3 a	1.01 cde	60.3 abc	18.3 a	1.08 cd
	**100% + 200**	60.3 a	10.0 ab	0.88cde	67.3 ab	15.0 a	1.09bc	58.0 ab	16.3 a	0.94 e	66.7 ab	19.0 a	1.06 cde
	**100% + 300**	57.7 ab	12.0 a	0.89 bcde	70.3 a	14.0 ab	1.11 b	59.0 a	13.7 ab	0.98 de	70.0 a	15.3 b	0.80 def
	**80% + con**	51.0 bc	7.0 cd	0.80 ef	54.7 cde	10.0 cd	0.66 e	42.0 def	9.0 cde	0.61 f	51.0d	11.3 cd	0.75 ef
	**80% + 100**	52.0 abc	8.0 bcd	0.99 bcd	60.0 bcd	11.7abc	0.96 cd	46.3 cde	10.0 cde	1.07 bcde	57.0 bc	12.3 cd	1.19 c
	**80% + 200**	56.7 ab	9.3 abc	1.01 abc	63.7 abc	13.0abc	1.15 b	48.0 cd	11.3 bc	0.95 de	60.7 abc	13.0 bc	1.16 c
	**80% + 300**	55.0 ab	12.0 a	1.05 ab	66.3 ab	14.3 ab	1.19 b	49.3 bcd	13.3 ab	1.19 abc	63.3 abc	15.0 b	1.29 c
	**60% + con**	38.0 e	6.3 d	0.97 f	47.0 e	8.0 d	0.93 d	35.0 f	7.3 e	1.03 bcde	44.0 d	10.0 d	1.15 c
	**60% + 100**	42.0 de	6.7 cd	1.01 abc	53.7 de	9.7 cd	0.95 d	38.7 ef	7.7 de	1.21 ab	52.0 cd	11.7 cd	1.35 bc
	**60% + 200**	44.0 cde	9.3 abc	1.16 a	55.0 cde	11.0bcd	1.38 a	47.0 cde	9.0 cde	1.37 a	55.0 bcd	12.3 cd	1.67 ab
	**60% + 300**	49.7 bcd	8.0 bcd	1.05 ab	58.3 bcd	12.0 bc	1.38 a	47.4cde	8.0 de	1.15 bcd	58.0 abc	12.0 cd	1.71 a
**L.S.D (0.05) = **													
**A**		**4.5**	**1.4**	**0.08**	**4.7**	**1.7**	**0.07**	**4.6**	**1.6**	**0.10**	**6.3**	**1.3**	**0.16**
**B**		**5.2**	**1.6**	**0.96**	**4.4**	**2.0**	**0.08﻿**	**5.3**	**1.8**	**0.12**	**7.2**	**1.5**	**0.19**
**AXB**		**8.9**	**2.7**	**0.17**	**9.4**	**3.3**	**0.13**	**9.2﻿**	**3.0**	**0.20**	**12.5**	**2.5**	**0.32**

Different letters within columns indicate significant differences (*P* < 0.05) of variation

*Irr.* Irrigation levels (% of ET_o_), *K.* Kaolin rates (ppm), *PH* Plant height(cm), *NB* Number of shoots, *LT* Leaf thickness (mm)

Foliar application led to a significant increase in all studied characteristics. Kaolin rate at 300 ppm enhanced plant height by 30.55%, shoot number by 26.38%, and leaf thickness by 29.25% as compared with the untreated plants grown under the same irrigation level at 60% based on ET_o_ in both 1st and 2nd cut during both seasons. These results are in agreement with the findings at [34] on *Physalis peruviana *kaolin application enhanced plant height, total dry mass in water-stressed cape gooseberry plants, and lowered leaf temperature. This is due to the foliar application of kaolin on *Cucurbita pepo* L. plant increasing the moisture status of treated plants during water deficit conditions [35]. Furthermore, kaolin increases the absorption of essential elements, such as potassium, phosphorus, and nitrogen, which are required for plant growth. Consequently, kaolin enhanced the activities related to plant productivity [15] on Maize plants.

### Effect of water deficit and kaolin rates on geranium yield

Data illustrated in Table 5 showed the effect of irrigation treatments and spraying with kaolin on fresh herb yield/plant (g), dry weight (g), and yield of fresh herb (ton)/fed. Data showed that, water deficit had a negative effect on biomass accumulation similar trends to vegetative growth parameters. Precisely, water deficit at 60% based on ET_o_ led to a decrease in yield/fed. by 27.77% in plants grown under 1st season conditions compared with full irrigated plants. These negative results could be attributed to all growth characteristics and biochemical processes resulting from the water stress disorders such as prevention of water and photo-assimilate translocation, photosynthetic capacity, and nutrient take-up [36] and [37] on tomato. Furthermore, this decay may be clarified by a diminish in the development of leaf cells or indeed by a lower rate of cell division in the plant, which in turn causes a decrease in dry matter and the generation of plant yield [38] and [39] on sweet pepper (*Capsicum annuum* L.)

**Table 5:** Effect of Irrigation levels and kaolin rates on yield of geranium (*Pelargonium graveolens*) plants

		**First Season (2022)**						**Second Season (2023)**					
**Treatments**		**First cut**			**Second cut**			**First cut**			**Second cut**		
		**FW (g)**	**DW (g)**	**FW (ton/f.)**	**FW (g)**	**DW (g)**	**FW (ton/f.)**	**FW (g)**	**DW (g)**	**FW (ton/f.)**	**FW (g)**	**DW (g)**	**FW (ton/f.)**
**Irr** **(A)**	**100% (con.)**	784.7 a	130.6 a	16.5 a	1030.9 a	167.5 a	21.7 a	819.1 a	131.0 a	17.2 a	1012.5 a	167.5 a	21.3 a
	**80%**	675.0 b	118.4 a	14.2 b	843.8 b	148.0 b	17.7 b	592.5 b	103.2 b	12.4 b	849.0 b	148.7 ab	17.8 b
	**60%**	581.0 c	107.9 a	12.2 c	716.2 c	134.6 b	15.3 c	488.0 c	89.7 b	10.3 c	691.7 c	127.6 b	14.0 c
**K** **(B)**	**Control**	616.6 c	89.8 c	13.0 c	770.8 b	112.0 c	16.2 b	558.3 c	80.9 c	11.7 c	772.3 b	112.2 c	16.2 b
	**100**	649.1bc	110.9bc	13.6 bc	811.3 b	138.5 b	17.0 b	597.0 bc	102.0 b	12.5 bc	805.6 ab	137.5 bc	16.2 b
	**200**	734.4 a	134.4 ab	15.4 a	904.6 a	168.2 a	19.3 a	658.3 ab	119.5 a	13.8 ab	910.8 a	166.3 ab	19.1 a
	**300**	720.9 ab	140.7 a	15.1 ab	967.8 a	181.5 a	20.3 a	719.2 a	129.6 a	15.1 a	915.6 a	175.6 a	19.2 a
**Interactions** **(A X B)**	**100% + con**	722.5 bc	93.6bcd	15.2bc	903.1 b	116.9 e	19.0 b	698.6 cd	92.2defg	14.7bcd	903.1 bc	116.9 cd	19.0bc
	**100% + 100**	729.2abc	125.7abcd	15.3abc	911.4 b	157.1 cd	19.1 b	786.6 bc	134.3 ab	16.5 bc	911.4 bc	157.2 abc	19.1 bc
	**100% + 200**	874.4 a	155.2 a	18.4 a	1092.9 a	194.0 ab	23.0 a	834.6 b	148.2 a	17.5 ab	1092.9ab	193.2 ab	23.0 ab
	**100% + 300**	812.8 ab	147.9 a	17.1 ab	1216.0 a	201.1 a	25.5 a	956.5 a	149.4 a	20.1 a	1142.7 a	202.6 a	24.0 a
	**80% + con**	621.9cde	93.4bcd	13.1cde	777.4bcd	116.8 e	16.3 bcd	565.1 e	84.0efg	11.9 de	798.7cde	119.8 cd	16.8 cd
	**80% + 100**	678.2bcd	116.5abcd	14.2bcd	847.8 bc	145.6 d	17.8 bc	573.7 e	98.5cdef	12.1 de	847.2 cd	145.6 bcd	17.8 c
	**80% + 200**	689.7 bc	123.8abcd	14.5bc	862.2 bc	154.9 cd	18.1 bc	585.5 de	104.4 de	12.3 d	862.2 c	154.6abc	18.1 c
	**80% + 300**	710.3bc	139.8 ab	14.9 bc	887.8 bc	174.8 abc	18.6 bc	645.8 de	125.9abc	13.6 cd	887.8 c	174.8 ab	18.6 c
	**60% + con**	505.5 e	82.6 d	10.6 e	631.8 e	102.3 e	13.3 e	411.1 f	66.5 g	8.6 f	615.2 e	99.9 d	12.9 de
	**60% + 100**	539.8 de	90.4 cd	11.3 de	674.8 de	112.9 e	14.2 de	430.8 f	73.2 fg	9.1ef	658.1 de	109.7 cd	11.7 e
	**60% + 200**	639.0cde	124.2abcd	13.4cde	758.7cde	155.7 cd	16.8cde	554.8 e	105.8cde	11.7def	777.4cde	151.3abcd	16.3 cd
	**60% + 300**	639.5cde	134.6abc	13.4cde	799.4bcd	167.6bcd	16.8bcd	555.2 e	113.5bcd	11.7def	716.2cde	149.3 bcd	15.0 cde
**L.S.D (0.05) = **													
**A**		**72.8**	**24.0**	**1.5**	**67.7**	**13.7**	**1.4**	**58.1**	**14.2**	**1.59**	**98.7**	**26.3**	**2.1**
**B**		**84.1**	**27.7**	**1.8**	**78.2**	**15.8**	**1.6**	**67.1**	**16.3**	**1.83**	**114.0**	**30.3**	**2.4**
**AXB**		**145.6**	**48.0**	**3.1**	**135.4**	**27.4**	**2.9**	**116.2**	**28.3**	**3.18**	**197.4**	**52.6**	**4.2**

Different letters within columns indicate significant differences (*P* < 0.05) of variation

*Irr.* Irrigation levels (% of ET_o_), *K.* Kaolin rates (ppm), *FW* Fresh weight (g/plant), *DW* Dry weight (g/plant), *FW (ton/fed)* Fresh weight (ton/fed)

Foliar application of kaolin tended to have a significant effect on biomass accumulation as well as dry matter of geranium plant and alleviate the adverse effects of water deficit, corresponding to an increase by 61.61% on plant dry matter and by 26.26% on plant yield treated by kaolin at 300ppm rate grown under higher water deficit. These findings align with earlier research [35] which noted that numerous plants generate less total leaf area at severe water stress. This is due to the reduced rate of cell division and development under osmotic stress and the reduction of turgor pressure and leaf loss results from the generation of ethylene and ABA. kaolin foliar application increased the moisture status, which led to enhanced cell division, nitrogen metabolism, enzymatic activity, and protein content of treated plants relative to untreated plants during water stress conditions. Also [40], Found that use kaolin spraying on *Zea mays* L. at intervals to reduce water stress and enhance plant nutrient uptake. The substantial increase in yield and economics over alternative mulching materials can be attributed to the improved physical condition of the soil, which favorably increased nutrient uptake by the crop by supplying a sufficient amount of N, P, and K through a steady and slow rate of nutrient release. Applying anti-transpirants improved metabolic, enzymatic, and protein synthesis under drought stress, which may have improved the harvest index by preserving relative plant hydration and lowering transpiration water loss. Plant water potential was increased during flower development by applying kaolin and coir pith. By reducing transpiration loss and increasing plant water potential during flower development, kaolin increased maize output. In a semi-arid area, coir pith mulching produced a greater grain yield than all other mulches combined.

### Essential oil productivity

#### Effect of water deficit and kaolin rates on oil content and geranium oil yield

Data in Table 6 showed that, essential oil (E.O.%) contents was increased in geranium herb as plant was subjected to water deficit. Maximum increased obtained in herb as plant irrigated with 60% based on ET_o_ by 23.08% followed by plant irrigated with 80% based on ET_o_ increased by 8.26% compared with regular irrigation. Similar findings were noted by [41], on *Thymus daenensis*. and [42] on (*Coriandrum Sativum* L.). On contrary, the lowest essential oil yield/fed. obtained from plants which irrigated with 60% based on ET_o_ reduction by 10.73% followed by 80% based on ET_o_ reduction by 8.20% compared with plants which irrigated with 100% based on ET_o_ [43] on thymus plants and [10] on Sweet basil plants, mentioned that, the decrease in biomass productivity was probably the reason for decrease the essential oil yield during the water deficit.

**Table 6:** Effect of Irrigation Levels and kaolin concentrations on essential oil contents of geranium (*Pelargonium graveolens*) plants

		**First Season (2022)**						**Second Season (2023)**					
**Treatments**		**First cut**			**Second cut**			**First cut**			**Second cut**		
		**E.O. %**	**oil/p (ml)**	**oil/F (L)**	**E.O. %**	**oil/p (ml)**	**oil/F (L)**	**E.O. %**	**oil/p (ml)**	**oil/F (L)**	**E.O. %**	**oil/p (ml)**	**oil/F (L)**
**Irr** **(A)**	**100% (con.)**	0.14 b	1.139 a	22.93 a	0.155 b	1.575 a	28.86 a	0.14 c	1.12 a	23.48 a	0.16 c	1.53 a	29.50 a
	**80%**	0.15 b	1.052 a	22.01 a	0.169 b	1.435 ab	27.18 a	0.16 b	0.93 b	19.55 b	0.17 b	1.43 a	27.75 a
	**60%**	0.18 a	1.036 a	21.87 a	0.190 a	1.395 b	26.26 a	0.18 a	0.89 b	18.67 b	0.20 a	1.40 a	26.83 a
**K** **(B)**	**Control**	0.13 b	0.799 b	16.89 b	0.146 c	1.119 c	20.57 c	0.14 b	0.77 b	16.26 b	0.16 b	1.12 b	21.97 b
	**100**	0.15 b	0.932 b	19.54 b	0.163 bc	1.319 b	24.75 b	0.15 b	0.88 b	18.52 b	0.16 b	1.31 b	24.45 b
	**200**	0.17 a	1.247 a	25.19 a	0.182 ab	1.664 a	31.28 a	0.17 a	1.10 a	23.01 a	0.19 a	1.66 a	32.56 a
	**300**	0.19 a	1.324 a	27.47 a	0.192 a	1.771 a	33.12 a	0.18 a	1.17 a	24.49 a	0.20 a	1.72 a	33.12 a
**Interactions** **(A X B)**	**100% + con**	0.12 d	0.863 de	18.21 de	0.137 e	1.260defg	23.54def	0.13 e	0.92 bc	19.37 bc	0.14 e	1.26 def	23.95 de
	**100% + 100**	0.13 cd	0.970 de	20.43 cde	0.150 cde	1.367cdef	25.87cde	0.14 de	1.10 ab	23.08 ab	0.15 de	1.35 cde	26.04bcd
	**100% + 200**	0.16bc	1.367 a	25.71 abc	0.157 cde	1.790 ab	32.61 ab	0.15cde	1.26 a	26.53 a	0.16 cde	1.79 a	33.91 a
	**100% + 300**	0.17 bc	1.357 a	27.36 a	0.167bcde	1.883 a	33.41 a	0.15 cde	1.19 a	24.95 a	0.17 cde	1.73 ab	34.08 a
	**80% + con**	0.13 cd	0.827 de	17.46 de	0.140 de	1.087 fg	19.94 f	0.15cde	0.84 cd	17.57 cd	0.16 de	1.09ef	22.27 de
	**80% + 100**	0.15 bcd	1.020 cd	21.13 bcde	0.167 bcde	1.440 cde	27.26bcd	0.15 cde	0.87 bcd	18.34bcd	0.17 cde	1.42 bcde	25.61cde
	**80% + 200**	0.14 cd	1.080bcd	22.60 abcd	0.177 bc	1.557 bcd	29.68abc	0.16cde	0.92 bc	19.39 bc	0.18 cd	1.55 abcd	30.94abc
	**80% + 300**	0.18 ab	1.280abc	26.84 ab	0.193 ab	1.657 abc	31.85 ab	0.18bc	1.09 ab	22.89 ab	0.19 bc	1.66 abc	32.19 ab
	**60% + con**	0.14 cd	0.707 e	15.00 e	0.160 bcde	1.010 g	18.23 f	0.14 de	0.56 e	11.83 e	0.17 cde	1.01 f	19.70 e
	**60% + 100**	0.15 bcd	0.807 de	17.06 de	0.173 bcd	1.150 efg	21.33 ef	0.16 cd	0.67 de	14.14 de	0.16 cde	1.14 ef	21.71 de
	**60% + 200**	0.20 a	1.293abc	27.25 ab	0.213 a	1.647 abc	31.55 ab	0.20 ab	1.10 ab	23.10 ab	0.22 ab	1.65 abc	32.83 a
	**60% + 300**	0.21 a	1.337 ab	28.02 a	0.217 a	1.773 ab	34.11 a	0.13 e	1.22 a	25.62 a	0.23 a	1.77 a	33.08 a
**L.S.D (0.05) = ** **A** **B** **AXB**		**0.02** **0.02** **0.04**	**0.138** **0.159** **0.276**	**3.07** **3.55** **6.15**	**0.018** **0.021** **0.036**	**0.163** **0.188** **0.325**	**2.78** **3.21** **5.56**	**0.02** **0.09** **0.03**	**0.12** **0.14** **0.25**	**2.60** **3.20** **5.20**	**0.02** **0.02** **0.03**	**0.17** **0.20** **0.34**	**3.17** **3.66** **6.33**

Different letters within columns indicate significant differences (*P* < 0.05) of variation

*Irr.* Irrigation levels (% of ET_o_), *K.* Kaolin rates (ppm), *E.O. %* Essential oil content, *oil/p* Oil Content /plant (ml), *oil/F* Oil yield /Fed. (L.)

Foliar application of kaolin rates on geranium plants, increased E.O. content oil content per plant (ml) and oil yield per fed. (L) significantly increased at the level of 200 and 300 ppm in both two-level water deficits. The highest increase by 57.14% was obtained at 300 ppm kaolin rate on plants grown under water deficit 60% based on ET_o_ compared with untreated plants in the same level water deficit. Moreover, yield per fed. (L) had significantly augmented increased. It increased by 117.86% compared with untreated plant grown under water deficit 60% based on ET_o_. These findings align with earlier research at [44] spraying kaolin on *Touriga-Nacional* contributed to the increased values of esters, alcohols, and volatile phenols compared with control. Also, these results are in agreement with those obtained by [45] on sweet basil who found spraying kaolin gave a significant increase in the oil percentage, oil content per plant and per fed. and harmony with [46] on *Ocimum basilicum* L. noted that, spraying with kaolin led to increased oil production. and increased the main components of the essential oil of basil.

#### Effect of water deficit and kaolin rates on oil chemical composition of *Pelargoniumgraveolens* plants

Table 7 presents the physiological reactions of the constituents present in geranium oil, which are affected by water deficit and application of kaolin. Total of thirteen main constituents were identified through the use of gas chromatography and mass spectrometry (G.C.M.) in geranium oil.

**Table 7:** Effect of irrigation levels and kaolin rates on essential oil constituents of *Pelargonium graveolens* (Average of two seasons 2022 and 2023)

Identified constituents (%)	Water levels based on ET_o_					
	**100%**		**80%**		**60%**	
	**Control**	**Kaolin**	**Control**	**Kaolin**	**Control**	**Kaolin**
α-pinene	0.71	0.24	0.41	0.30	0.24	0.16
Limonene	3.65	4.12	4.65	2.42	0.75	0.77
L-linalool	0.70	2.06	0.76	1.11	1.06	0.88
Nerol	9.08	nd	5.24	nd	nd	6.23
Rose oxide	8.12	0.10	1.18	7.17	5.01	0.98
Citronellol	28.95	27.90	21.90	23.50	25.76	24.69
Geraniol	11.41	13.72	14.42	17.41	20.99	23.45
Isogeraniol	2.00	1.15	5.82	2.32	2.13	2.79
Citral	1.15	1.60	2.79	3.64	0.20	1.67
Citronellyl formate	1.94	7.20	3.67	2.89	2.30	2.98
Geranyl acetate	0.55	0.05	3.75	3.44	1.55	1.79
10-epi-ҫ-eudesmol	12.44	9.08	12.30	11.82	14.87	11.00
Geranyl tiglate	1.27	1.32	2.99	5.09	2.90	4.76
**C/G ratio**	**2.54**	**2.03**	**1.52**	**1.35**	**1.23**	**1.05**

Kaolin Rates at 300 ppm

*nd *Not detected, *C* Citronellol *G* geraniol

The composition of geranium oil was significantly affected by the foliar application of kaolin. At every irrigation level, the relative proportions of different monoterpenes were altered. Deficit level and kaolin application rate had a substantial impact on reactions involving oxygenated monoterpenes, including geraniol, isogeraniol, citronellol, citronellylformate, citral, linalool, and geranyl acetate. Based on the combined effects of irrigation and kaolin levels, oxygenated constituents appeared as the most significant category among the oil constituents, accounting for between 47.60% and 60.20%. The ratios of geraniol to citronellol indicate the quality of geranium oil. Both of these compounds are known to be able to change into one another, with geraniol serving as a precursor to citronellol. This understanding was backed by earlier research by [4] and [47]. An essential metric is the C/G ratio, which has a range of 1 to 3. A ratio closer to 1 is frequently linked to a higher-quality product and yields the highest quality [4]. It's interesting to note that the current study discovered that higher water stress led to a lowest C/G ratio by decreasing the amount of citronellol and increasing geraniol. Nevertheless, this reduction was only noticed in the presence of 300 ppm kaolin.

The findings are in harmony with study by [48], who found that the chemical composition essential oil was altered by water stress circumstances that favor the development of secondary plant metabolites like essential oils [49]. Also discovered that there was a relationship between the frequency of irrigation and the composition of essential oils, with geraniol, the primary volatile component, declining as the percentage of water depletion increased.

### Anatomical study

**Fig. 1:**
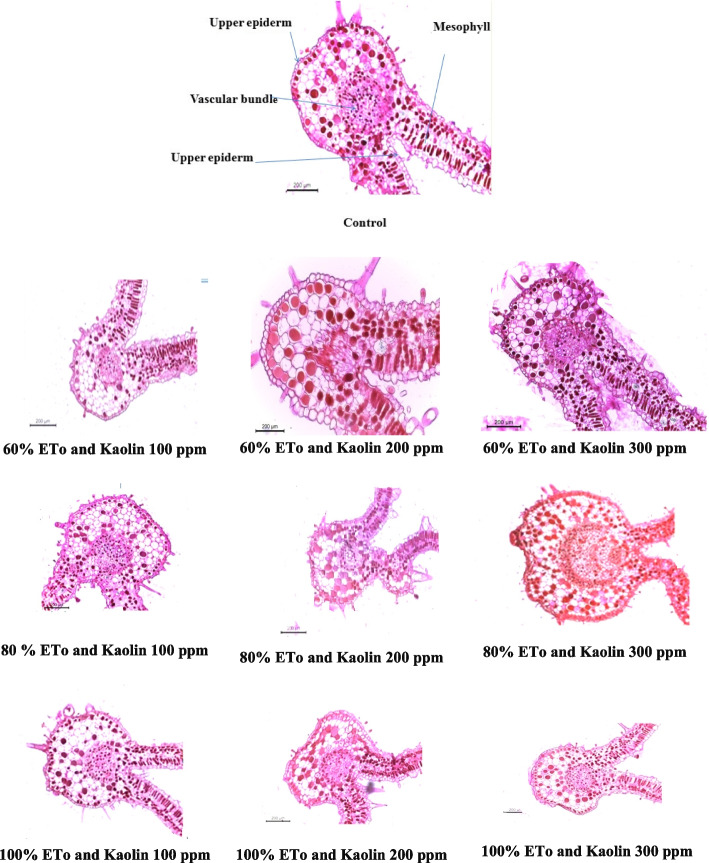
Transverse sections of Pelargonium graveolens leaf treated with draught and kaolin (10X)

The measurement of the anatomical parameters of *Pelargonium graveolensL* sand. Leaves subjected to drought deficit and kaolin treatment are shown in Fig. 1 and Table 8. The results indicate that the thickness of the upper epidermis showed a significant increase compared to the control group. More precisely, at 60% and 200 it reaches 53.7%. In contrast, the thickness of the lower epidermis only increased by 28.7% in treatment of 60% and 200 ppm.

**Table 8:** Effect of irrigation levels and kaolin rates on anatomical parameters of *Pelargonium graveolens* leaves (mm)

Characters (mm)	Water levels based on ET_o_									
	**100**				**80**			**60**		
					**Kaolin rates (ppm)**					
	**Control**	**100**	**200**	**300**	**100**	**200**	**300**	**100**	**200**	**300**
Upper epidermis thickness	23.784	26.918	36.570	15.833	30.940	26.735	20.610	31.249	38.745	23.076
Lower epidermisthickness	45.264	25.672	29.530	15.413	27.424	21.891	22.700	22.022	58..272	44.731
Palisade tissue thickness	52.888	55.680	54.223	48.678	92.508	37.208	59.492	72.238	108.679	57.760
Spongy tissue thickness	120.045	156.439	155.532	70.400	111.901	94.670	116.138	192.979	198.618	106.817
Mesophyll thickness	172.933	62.945	101.955	137.673	212.875	132.344	190.717	75.966	307.297	195.067
Midvein vascular bundle										
Width	239.631	288.736	297.787	241.664	744.510	179.936	348.257	219.993	289.898	289.213
Length	268.365	251.619	242.135	222.243	683.667	194.076	308.018	180.018	364.592	347.932

***mm***  **Millimeters**

Under the influence of irrigation conditions and different concentrations of kaolin, the palisade and sponge fabrics containing mesophyll showed significant improvement. In particular, at a kaolin concentration of 80% and under irrigation, the palisade fabric showed a remarkable improvement of 23%. Similarly, terry cloth showed a remarkable improvement of 77.7% when exposed to 100 ppm kaolin concentration and irrigation conditions. These results highlight the positive influence of irrigation and specific kaolin concentrations on the development and performance of mesophyll-containing tissues.

The width and length of the vascular bundle experienced a substantial increase of 210.6% and 154.7%, respectively, when subjected to concentrations of 80% and 100 ppm, as observed in studies conducted by [50] on *Triticum aestivum* [51] on *Solanum lycopersicum* L. Application of anti-transpirant under water deficit conditions resulted in improvement of all histological parameters. The use of three different anti-transpirant, at lower and higher concentrations, under water deficit conditions led to an increase in the thickness of the wheat leaf blade. This increase was attributed to thickening of the mesophyll tissue and vascular bundle, as well as a significant reduction in stomata opening at the upper and lower epidermis, compared to the control group [52] observed that anti transpirant do not cause permanent damage to the stomata mechanism and have specific effects on guard cells, without affecting other cells.

The aforementioned results provided evidence for the importance of kaolin, as discussed by [53, 54]. This function encompasses alterations of crucial morphological, physiological and biochemical processes through enhancement of radiation reflection. Initially, kaolin prevents the accumulation of thermal charge, thereby decreasing transpiration, while maintaining relatively high stomatal conductance.

Drought deficit has been shown to have obvious effects on the histological composition of plants, as shown in various experiments. Leaves of 'Chemlali' cultivar showed a significant increase in the thickness of spongy parenchyma and upper palisade under water deficit. The thicker palisade parenchyma in leaves may indicate to more CO2 fixation sites, while the thicker spongy parenchyma may facilitate more rapid diffusion of CO2 to these sites [55, 56] suggest that a decrease in water content in the plant body promotes cell wall strengthening and reduces turgor pressure. In the study [57], in agreement to negative impact of draught to plant tissue that led to decrease in lamina thickness and drought stress may decrease vessel diameter ris considered as an adaptation mechanism that protect plants from water deficiency caused by leaf transpiration and affects the conductance of CO2 diffusion that play acrucial role to avoid cavitation. Moreover, reductions in metaxylem diameter and vascular bundle size are normally in plant exposed to drought stress.

### Effect of water deficit and kaolin rates on photosynthetic pigment and carotenoid content

Chlorophyll a (6.93 mg/g FW) and chlorophyll b (3.00 mg/g FW) concentrations recorded the value highest in plants irrigated with 100% of ET_o_ combined with the highest rate of kaolin (300 ppm), (Table 9). Furthermore, when 100% of ET_o_ was combined with the treatment of kaolin at 200 ppm, a notable level of kaolin carotenoid content (4.44 mg/g FW) was noted. Conversely, the lowest levels of kaolin (100 ppm) in combination with 60% ET_o_ irrigation resulted in the lowest quantities of chlorophyll a (1.31 mg/g FW) and chlorophyll b (0.08 mg/g FW). Furthermore, the combination of 100 ppm spraying kaolin and 80% of ET_o_ irrigation resulted the lowest concentration of carotenoid (0.35 mg/g FW). The geranium plant experienced a decrease in chlorophyll and carotenoid content due to the suppression or degradation of chlorophyll biosynthesis caused by water deficit. As the duration of drought deficit in plants prolonged, the pigment content decreased significantly. However, the application of kaolin had a contrasting effect, leading to a significant increase in the chlorophyll and carotenoid content of the leaves. This finding is consistent with previous studies conducted on walnut [56] and grapevine [57]. On Loss of chlorophyll as a result of photo oxidation and subsequent oxidative damage is one effect of drought stress [58]. According to [15] on maize plant decrease in chlorophyll content, photosynthetic, net photosynthetic rate, and transpiration rate can be viewed as a sign of oxidative stress. while, kaolin led to increased photosynthetic pigments. This shows that the plants are more resilient to drought stress, which could lead to a higher energy efficiency in photosynthetic process. Same result as indicated by [14] on *Corylus avellana***.** According to [59] on *Amaranthus tricolor's* pigment content gradually decreased as drought stress increased, which is in line with our observations. Similar decreases in pigment concentration were also noted by [60] in peanuts and by [9] in *Mentha pulegium.*

**Table 9:** Effect of irrigation levels and kaolin rates on chlorophyll (a, b) and carotenoid concentration (mg g-^1^ F.W.) of *Pelargonium graveolens *plant

Pigment	Water levels based on ET_o_											
	**100**					**80**				**60**		
						**Kaolin rates (ppm)**						
	**0.0**	**100**	**200**	**300**		**100**	**200**	**300**		**100**	**200**	**300**
**Total Chl**	**7.06 ± 0.80e**	**14.36 ± 4.21b**	**14.42 ± 3.33b**	**17.15 ± 3.34a**		**5.78 ± 1.34ee**	**9.97 ± 2.76c**	**12.92 ± 3.46d**		**1.74 ± 1.01 g**	**4.61 ± 1.66f**	**4.82 ± 1.78f**
**Chl a**	**5.32 ± 3.07b**	**5.22 ± 3.01b**	**6.81 ± 3.93a**	**7.09 ± 4.09a**		**2.53 ± 1.46de**	**3.34 ± 1.93 cd**	**4.07 ± 2.35c**		**1.31 ± 0.76f**	**1.73 ± 1.00ef**	**2.97 ± 1.72d**
**Chl b**	**2.07 ± 0.04b**	**1.72 ± 0.12c**	**2.24 ± 0.12b**	**3.00 ± 0.29a**		**0.34 ± 0.02de**	**0.35 ± 0.02de**	**0.48 ± 0.02d**		**0.08 ± 0.01e**	**0.09 ± 0.01e**	**0.3 ± 0.06de**
**Caro**	**0.71 ± 0.06e**	**3.40 ± 0.01b**	**4.44 ± 0.02a**	**2.86 ± 0.02c**		**0.35 ± 0.01e**	**4.22 ± 0.34a**	**2.40 ± 0.23 cd**		**2.40 ± 0.12 cd**	**2.31 ± 0.17d**	**2.77 ± 0.17 cd**

Data are the means ± SE of three different experiments with three replicated measurements; different letters within a column indicate significant differences (*P* < 0.05) of variation

*Chl.* Chlorophyll, *Chl a* Chlorophyll a, *Chl b* Chlorophyll b, *Caro.* Carotenoid. (mg g^−1^ F.W.)

### Effect of irrigation levels and kaolin rates on antioxidant enzymes activities

Table 10 presents the results of the investigation into the impact of water deficit treatments and different levels of kaolin on metabolizing enzymes. The study focused on leaves of *Pelargonium graveolens*, specifically analyzing the activity levels of SOD, POD and CAT in its leaves. The results indicated a notable increase in SOD and POD activities, while CAT activity showed a decline under drought treatment. However, the negative effects of water deficit were mitigated by the application of kaolin to the plant.

**Table 10:** Effect of irrigation levels and kaolin rates on antioxidant enzymes activities (U/g/ FW/hour) of *Pelargonium graveolens* plant

enzymes	Water levelsbased on ET_o_											
	**100**					**80**				**60**		
						**Kaolin rates (ppm)**						
	**0.0**	**100**	**200**	**300**		**100**	**200**	**300**		**100**	**200**	**300**
**SOD**	**0.3 ± 0.03def**	**0.38 ± 0.02cde**	**0.23 ± 0.01ef**	**0.15 ± 0.03f**		**1.28 ± 0.03a**	**0.49 ± 0.02c**	**0.45 ± 0.03 cd**		**1.35 ± 0.09a**	**1.05 ± 0.03b**	**0.9 ± 0.12b**
**CAT**	**0.05 ± 0.01de**	**0.5 ± 0.06b**	**0.92 ± 0.12a**	**0.95 ± 0.03a**		**0.15 ± 0.02cde**	**0.17 ± 0.01 cd**	**0.23 ± 0.06c**		**0.02 ± 0.01e**	**0.11 ± 0.01 cde**	**0.17 ± 0.02 cd**
**POD**	**0.18 ± 0.01 cd**	**0.19 ± 0.02 cd**	**0.18 ± 0.02 cd**	**0.13 ± 0.02d**		**0.25 ± 0.03bc**	**0.21 ± 0.01 cd**	**0.2 ± 0.2 cd**		**0.34 ± 0.02a**	**0.31 ± 0.06ab**	**0.3 ± 0.03ab**

Data are the means ± SE of three different experiments with three replicated measurements; different letters within rows indicate significant differences (*P* < 0.05) of variation

*SOD* Superoxide dismutase, *POD* Hydrogen peroxidase, *CAT* Catalase

In Table 10 droughtness of soil preferentially increased the activities of superoxide dismutase (SOD) and peroxidase (POD), which increased the rate from 0.3 to 1.35 and from18 to 34 U/g/F.W./hour, respectively, while catalase activity (CAT) declined. These results are in complete agreement with those reported by [61] mention that, the upregulation of antioxidant enzymes represents an important marker for drought stress. In the cell, the production and scavenging of ROS is strictly controlled by an efficient and versatile scavenging system. The antioxidant defense system comprises enzymes such as including CAT, and SOD. While [62] found that on *Oryza sativa* L. CAT and SOD were an important enzyme used to eliminate H_2_O_2_. With the prolongation of drought time, observed that the CAT activity increased in leaves. While POD activity increased at the beginning of the drought and then seemed to enter a platform stage. The same results were absolved at [63] on wheat.

The result obtained that foliar application of kaolin increases the activity of antioxidant enzymes. The results are in agreement with [64], from studies on apple kaolin helps plants withstand environmental stress by stimulating their enzyme systems and preserving their cell membrane. spraying kaolin on apple leaves enhanced the activity of antioxidant enzymes such as glutathione reductase, superoxide dismutase, catalase, and ascorbate peroxidase during drought stress. And in align with [14], who indicated that. kaolin applications on hazelnut increased proline and antioxidant enzymes. Conversely, there was a decrease in protein concentration, H_2_O_2_ level, and lipid peroxidation.

### Effect of irrigation levels and kaolin rates on water use efficiency (WUE)

**Fig. 2:**
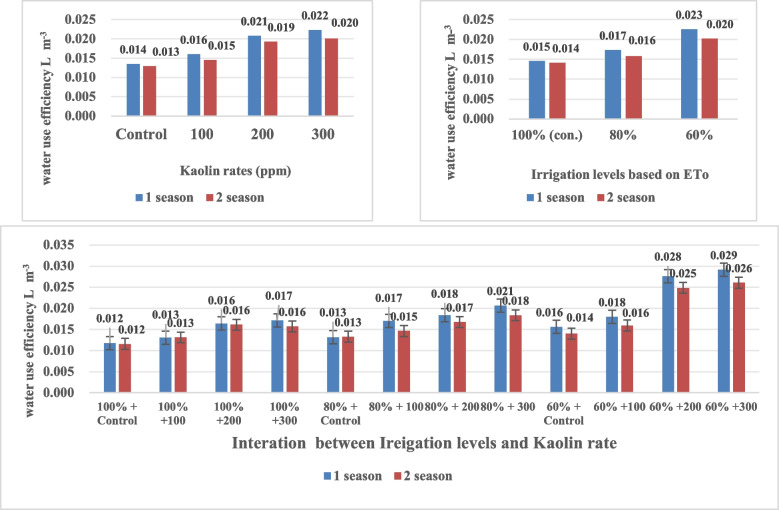
Effect of irrigation levels and Kaolin rate on water use efficiency in total oil yield of *Pelargonium graveolens* during two season

Results of geranium WUE are affected by water deficit and kaolin rate as shown in Fig. 2. The highest values of WUE were obtained by 60% of ET_o_ (0.23 and 0.20 L/m^3^) in 1^st^ and 2^nd^ seasons, respectively. These results are in agreement with [65] who mentioned that both WUE and yield can be improved under drought deficit.

Kaolin at rates of 200 and 300 ppm showed an improving WUE due to reducing losses of water through evaporation by closed leaves stomata during different growth stages. According to, improved transportation of all soluble substances leads to better growth and yield. The same results were obtained at [34] on gooseberry plants.

## Conclusion

It concluded that geranium plant growth characteristics, fresh herb yield/fed, and essential oil yield/fed gradually decreased as the water deficit increased. Comparing fresh herb yield/fed and essential oil yield/fed to regular irrigation, the biggest decreases occurred at a water level of 60% based on ET_o_, which was 27.77% and 0.73%, respectively. However, when the water level dropped, plants that were irrigated 60% of the time according to ET_o_ showed increases in leaf thickness and E.O.%. of 36.73% and 23.08%, respectively. According to the findings, kaolin foliar application at 200 and 300 ppm may help treat water deficit disorders by improving anatomical features. Compared to the untreated plant group, the upper epidermis' thickness considerably increased. More precisely, at 60% and 200, it reaches 53.7%. Additionally, the thickness of the lower epidermis increased by 28.7%. It has been shown that irrigation and specific kaolin concentrations improve the development and functionality of mesophyll tissues. Furthermore, kaolin dramatically raises the leaves' carotenoid and chlorophyll contents. And kaolin makes antioxidant enzymes more active. Additionally, when compared to untreated plants grown under a 60% water deficit based on ET_o_, kaolin spraying enhances the quality of essential oil by increasing geraniol at the expense of citronellol. This results in a 35% increase in water use efficiency and a yield/feed of essential oils of over 117.86%.

## Recommendation

Apply water deficit treatments one month before harvest and from the flower bud’s initiation phase to avoid a decrease in the vegetative yield and benefit from the increase in the oil percentage and quality resulting from the water deficit.

## Suggestions to the future

We need to conduct further research using kaolin in a different form (nano form), study it at the molecular level to understand gene expression, use the photosynthesis parameters determined by the Licor system, and apply it to other stressors.

## Data Availability

Data can be made available upon reasonable request from the corresponding author (M.A.E.A.) maekaoud@gmail.com.
